# Cross-Reactive Bactericidal Antimeningococcal Antibodies Can Be Isolated From Convalescing Invasive Meningococcal Disease Patients Using Reverse Vaccinology 2.0

**DOI:** 10.3389/fimmu.2018.01621

**Published:** 2018-07-16

**Authors:** Fadil A. Bidmos, Simon Nadel, Gavin R. Screaton, J. Simon Kroll, Paul R. Langford

**Affiliations:** ^1^Section of Paediatrics, Department of Medicine, Imperial College London, London, United Kingdom; ^2^St. Mary’s Hospital, Paddington, London, United Kingdom

**Keywords:** *Neisseria meningitidis*, invasive meningococcal disease, reverse vaccinology 2.0, human monoclonal antibodies, vaccines

## Abstract

The threat from invasive meningococcal disease (IMD) remains a serious source of concern despite the licensure and availability of vaccines. A limitation of current serogroup B vaccines is the breadth of coverage afforded, resulting from the capacity for extensive variation of the meningococcus and its huge potential for the generation of further diversity. Thus, the continuous search for candidate antigens that will compose supplementary or replacement vaccines is mandated. Here, we describe successful efforts to utilize the reverse vaccinology 2.0 approach to identify novel functional meningococcal antigens. In this study, eight broadly cross-reactive sequence-specific antimeningococcal human monoclonal antibodies (hmAbs) were cloned from 4 ml of blood taken from a 7-month-old sufferer of IMD. Three of these hmAbs possessed human complement-dependent bactericidal activity against meningococcal serogroup B strains of disparate PorA and 4CMenB antigen sequence types, strongly suggesting that the target(s) of these bactericidal hmAbs are not PorA (the immunodominant meningococcal antigen), factor-H binding protein, or other components of current meningococcal vaccines. Reactivity of the bactericidal hmAbs was confirmed to a single ca. 35 kDa protein in western blots. Unequivocal identification of this antigen is currently ongoing. Collectively, our results provide proof-of-principle for the use of reverse vaccinology 2.0 as a powerful tool in the search for alternative meningococcal vaccine candidate antigens.

## Introduction

*Neisseria meningitidis* is a major obligate human pathogen that frequently colonizes the nasopharynx asymptomatically, in a state known as carriage (1). Occasionally, invasive meningococcal disease (IMD) occurs, through invasion of pharyngeal tissues, proliferation in blood (meningococcal septicemia), and crossing of the blood–brain barrier leading to meningitis (2, 3). More than 70,000 cases of IMD are reported annually worldwide with case fatality ratios between 5 and 15%, even with therapeutic intervention (4, 5). Debilitating neurological sequelae are common among survivors of IMD (6–8).

The use of currently available polysaccharide conjugate meningococcal vaccines has been effective against targeted serogroups (mainly serogroups A, C, W, and Y) within vaccinated populations (9, 10). For serogroup B strains, which account for more than 60% of IMD in the UK and Europe (11, 12), vaccine development has focused heavily on sub-capsular vaccine components owing to the unsuitability of the serogroup B capsule (13). One of these vaccines, 4CMenB (or Bexsero^®^), is a protein-based vaccine whose major components are the factor-H binding protein (fHbp) (variant 1.1), Neisserial heparin-binding antigen (NHBA variant 2), Neisserial adhesin A (NadA variant 3), and the detergent-extracted outer membrane vesicle component of the New Zealand epidemic strain (with PorA variant P1.4) (14). Like the polysaccharide conjugate vaccines, accruing data shows high effectiveness of 4CMenB (15). However, we are seeing a gradual recrudescence of carriage and disease to pre-vaccine levels through vaccine-driven strain replacement (16–19). In addition, there are concerns that the changing epidemiology of IMD (20–22) may lead to a significant reduction in the efficacy of the vaccines in the long term. These limitations, coupled with the huge potential of the meningococcus to generate extensive antigenic diversity (leading to vaccine/immune escape) (23) justify the search for novel vaccine candidate antigens.

Preclinical vaccine development methods are enriched by detailed analysis of the human immune response to etiological agents of infectious diseases. For example, with the development of high-throughput technologies, deep sequencing of the gene segments encoding the variable regions of antibody heavy (V_H_) and light (V_L_ = κ or λ) chains in a given B cell repertoire is providing valuable information useful in understanding adaptive immunity to infections, autoimmunity, and malignancies (24, 25). Identifying the targets of antibodies of interest by cloning and *in vitro* expression of V_H_ and V_L_ chains of B-cell antibodies is a powerful approach, which can be utilized to inform on the functional immunogenicity of both known and novel antigens. The use of this approach, termed reverse vaccinology 2.0 (26), in the cloning of neutralizing human recombinant monoclonal antibodies [human monoclonal antibodies (hmAbs)] from patients convalescing from viral infectious diseases is well documented; the first studies in the use of reverse vaccinology 2.0 focused on the isolation and functional characterization of antibodies targeting the dengue, HIV, and influenza viruses (27–29). The power of the approach lies in the expression of paired V_H_ and V_L_ regions from individual plasmablasts or memory B cells; the output being the expression of hmAbs mimicking natural V_H_ + V_L_ combinations induced in the host.

Because of the transience of peak plasmablast circulation [reviewed in Ref. (30)] and the higher incidence of IMD among infants and toddlers (placing a limitation on blood sample volume), we aimed to assess whether reverse vaccinology 2.0 could be employed in the discovery of novel meningococcal antigens of vaccine potential. In this brief research report, we will outline findings relating to the following aims: (i) whether cross-reactive antimeningococcal hmAbs targeting surface proteins could be cloned from patient samples; and (ii) if these hmAbs possessed bactericidal activity against a wide panel of strains, specifically those not covered by the protein-based meningococcal vaccines.

## Materials and Methods

### Ethics Statement and Study Participants

Studies with human blood samples were approved by the London—Fulham Research Ethics Committee (Ref.: 11/LO/1982). Informed written consent was obtained from patients or their representatives in accordance with the Declaration of Helsinki. Patients were recruited following admission to the Imperial Healthcare (St. Mary’s Hospital) Paediatric Intensive Care Unit (PICU), London, UK.

Patient SM-P02 was a 7-month-old baby who presented with fever, irritability, and reduced oral intake. Rapidly spreading petechial rash was detected a few hours after arrival at the hospital. Blood cultures confirmed meningococcal septicemia. Molecular typing of the isolate, M14-240312, revealed that it was a serogroup B strain (MenB). A 4 ml blood sample was collected 7 days post-admission to St. Mary’s Hospital PICU, following confirmation of negative blood cultures.

### Bacterial Strains

Assays performed with whole bacteria or cell lysates involved patient isolates M14-240312 and a 16-strain meningococcal panel reflecting the current genetic epidemiological prevalence in the UK (obtained from the UK Meningococcal Reference Unit, Public Health England, Manchester) (Table 1). This 16-strain panel includes 10 strains genotypically mismatched for all the major 4CMenB antigens. Typing information was obtained from the *Neisseria* MLST database hosted on https://pubmlst.org/neisseria/. Meningococci were routinely grown on 5% horse blood agar or in brain heart infusion broth supplemented with 5% Levinthal’s.

**Table 1:** MenB strains composing the 17-strain screening panel.

S/N	Isolate	PorA		FetA VR	MLST	4CMenB antigen type		
		VR1	VR2			Factor-H binding protein	NHBA	NadA^a^
1	M14-240312	12-1	13-1	5-5	41/44	2.19	2	–
2	MC58	7	16-2	1-5	32	1.1	3	110
3	M07-240646	22	9	5-12	269	2.19	17	–
4	M07-240657	5-1	10-4	5-5	213	3.294	18	–
5	M07-240669	17-1	23	5-2	41/44	2.19	31	–
6	M07-240679	19	15	ND^b^	269	2.19	17	–
7	M07-240680	22	14-6	ND	41/44	2.19	43	–
8	M07-240909	22	14	5-5	213	2.19	18	–
9	M08-240014	22	9	ND	269	2.68	17	–
10	M08-240107	22	9	ND	269	3.59	17	–
11	M08-240164	22	14	ND	213	3.45	18	–
12	M08-240276	22	14	5-5	213	3.45	18	–
13	M10-240474	19-1	15-11	3-9	269	1.15	21	–
14	M10-240476	22	14	5-5	213	1.110	18	–
15	M10-240480	7-2	4	1-5	41/44	1.4	2	–
16	M11-240016	7	16	3-3	32	1.62	3	100
17	M11-240123	5-1	2-2	5-8	11	2.22	20	–

*^a^Dash (–) denotes absence of NadA in isolate*.

*^b^ND denotes no data, i.e. no typing information available for this antigen*.

### Cell Sorting, cDNA Synthesis, and V_H_/V_L_ Cloning

Peripheral blood mononuclear cells (PBMCs) were extracted from a 4 ml blood sample using a density gradient centrifugation method (Leucosep) and sorted singly into individual wells of 96-well plates containing catch buffer (10 mM Tris pH 8.0, 10 U RNAsin) as previously described (31). PBMCs were incubated with a cocktail of anti-human monoclonal antibodies targeting CD3 (PerCP/Cy5.5), CD14 (PerCP/Cy5.5), CD19 (RPE), CD20 (PerCP/Cy5.5), CD27 (FITC), CD38 (APC), and CD56 (PerCP/Cy5.5) for 30 min on ice, in the dark. Stained PBMCs were analyzed using a BD FACSAria III cell sorter. Plasmablasts were gated as follows: CD3^−^, CD14^−^, CD19^+^, CD20^−^, CD56^−^, CD27^high^, and CD38^high^. A single freeze-thaw cycle was used to lyse cells and release RNA. cDNA was synthesized from released RNA using the QIAGEN OneStep RT-PCR kit, as per manufacturer’s protocols. A nested PCR was used to amplify the V_H_ and V_L_ regions; primers contained restriction endonuclease sites, which facilitated cloning into respective AbVec-IgH (*AgeI* + *SalI*), AbVec-Igκ (*AgeI* + *BsiWI*), or AbVec-Igλ (*AgeI* + *XhoI*) expression vectors.

### Expression of hmAbs in Human Embryonic Kidney (HEK-293) Cells

HEK-293 cells were transiently transfected with cognate plasmid pairs using polyethyleneimine as transfection agent, as previously described (31). Culture supernatants were harvested after 72 h. HmAbs were purified from culture supernatants with single-use Protein G spin columns (Ab SpinTrap, GE Healthcare), as per manufacturer’s instructions.

### Assessment of hmAb Specificity

HmAb reactivity to surface-bound meningococcal antigens was assessed using indirect cell-based ELISAs, as previously described (32). Briefly, inactivated bacterial whole cell suspensions were normalized to an OD600 of ~0.5. Whole cells (100 µl of cell suspensions) or 1 µg of 4CMenB in Carbonate-Bicarbonate buffer (Sigma-Aldrich, cat. no. C3041) were transferred into designated wells of flat-bottom polystyrene 96-well plates and incubated at 4°C overnight. Wells were subsequently incubated with 200 µl of blocking buffer (PBS, 0.05% Tween-20, 1% BSA) for 1 h at room temperature prior to addition of 100 µl of an appropriate dilution of hmAbs or plasma IgG. Following a 1-h incubation, wells were washed thrice to remove unbound antibodies. A 1:2,000 dilution of an anti-IgG alkaline phosphatase conjugate (Sigma-Aldrich, cat. no. A9544) was used to probe wells for 1 h at room temperature. Wells were washed five times before addition of a phosphatase substrate (Bio-Rad, cat. no. 172-1063) as per the manufacturer’s instructions. Signal detection was performed at OD405 using a microplate reader.

Further investigations into hmAb reactivity were performed using western blotting, as described elsewhere (33).

### Serum Bactericidal Activity (SBA) Assay

Functional activity of antimeningococcal IgG was determined using the standardized SBA assay (34). Briefly, 10^3^ CFU meningococci were incubated with IgG (hmAb or purified total plasma IgG) (final assay concentration of 25%) and exogenous human complement (25%) for 90 min in a humidified CO_2_ incubator at 37°C, with gentle shaking. IgG that effected a reduction in meningococcal CFU by >50% after 90 min compared to negative controls (complement only; IgG only) was considered to possess SBA activity.

## Results

### Induction of a Functional Immune Response in Patient SM-P02

**Figure 1:**
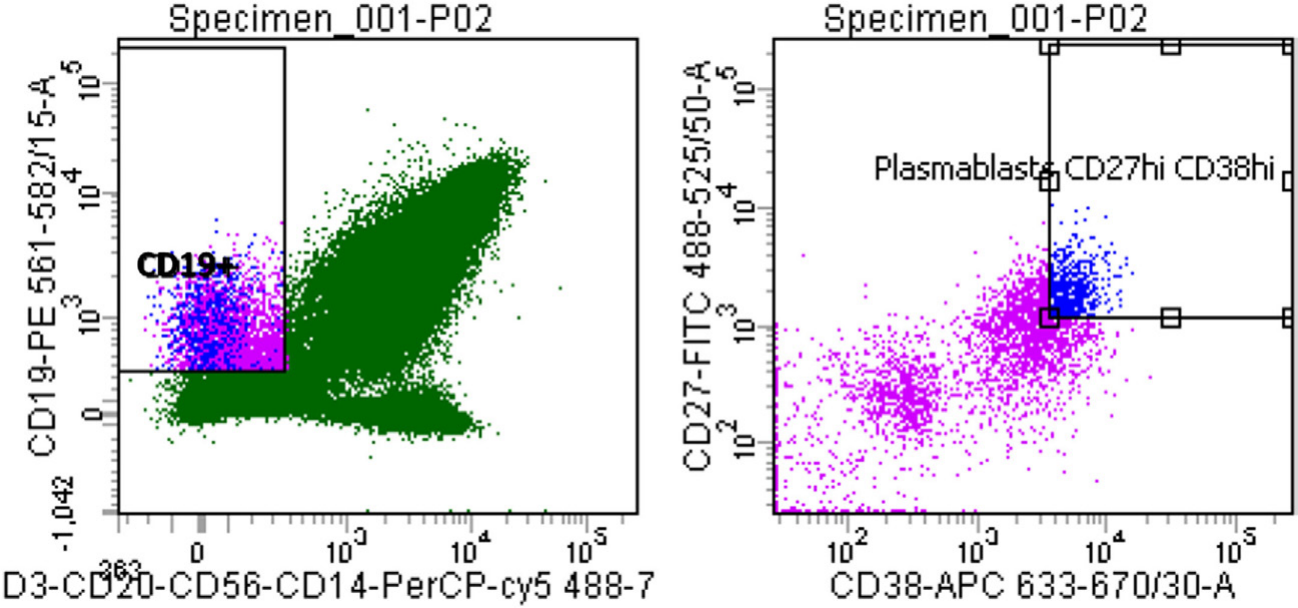
Quantitative analysis of patient SM-P02’s adaptive immune response to a MenB infection. Flow cytometry plots were gated for plasmablasts by CD3^−^, CD19^+^, then CD27^+/high^ and CD^38+/high^.

**Figure 2:**
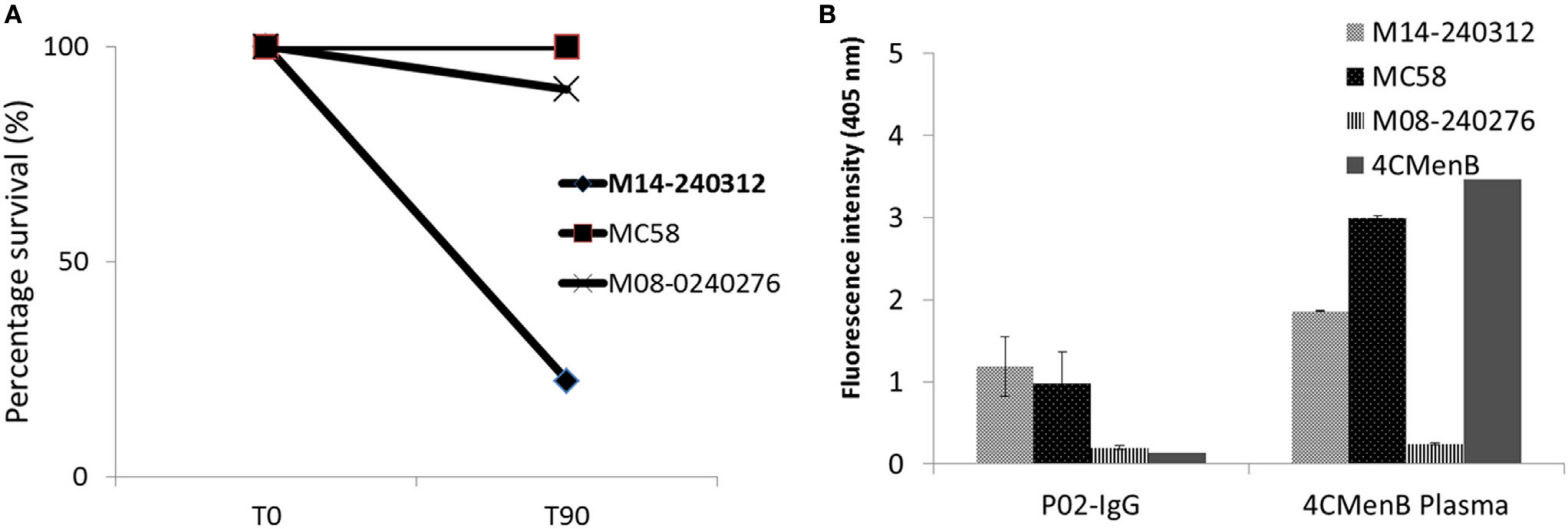
Qualitative analyses of patient SM-P02’s adaptive immune response to a MenB infection. **(A)** Serum bactericidal response of SM-P02 plasma IgG (final assay concentration of 192 µg/ml) to the infecting MenB strain, M14-240312. **(B)** Reactivity of SM-P02 plasma IgG to MenB strains and 4CMenB in ELISA. Cells normalized to OD600 of ~0.5 or 1 µg of 4CMenB were used as antigen in ELISAs (duplicate wells). Error bars represent SEM. Data were obtained from three biological assay replicates (*n* = 6).

Flow cytometric analysis showed that the circulating plasmablast population in patient blood was low, measured at 0.3% (Figure 1). To assess whether these plasmablasts represented a functional response to the meningococcal infection, total IgG purified from patient plasma was assayed for bactericidal activity. Purified total IgG, rather than plasma, was assayed because of the presence of administered antibiotics in patient plasma. Reactivity of purified SM-P02 IgG to the patient isolate (M14-240312) and two other strains (MC58, which expresses the fHbp variant 1.1 in 4CMenB; and M08-240276, which is mismatched for all 4CMenB antigens) was assessed in ELISAs prior to assessment of SBA activity against the three strains. SM-P02 IgG bound to the infecting isolate, M14-240312, in cell-based ELISAs with corresponding SBA activity against the isolate. Despite significant binding of SM-P02 IgG to MenB strains MC58 and M08-240276 in ELISAs, no bactericidal activity was discerned against these strains. Thus, the induction of a specific bactericidal response in the patient to M14-240312 was confirmed (Figure 2A). Furthermore, the antimeningococcal immune response in patient SM-P02 was exclusive of the antigenic components of 4CMenB as no reactivity of SM-P02 plasma IgG to the vaccine antigens was discerned in ELISAs, under the experimental conditions employed in this study (Figure 2B).

### *In Vitro* Cloning and Expression of Antimeningococcal Antibodies From Patient Plasmablasts

**Figure 3:**
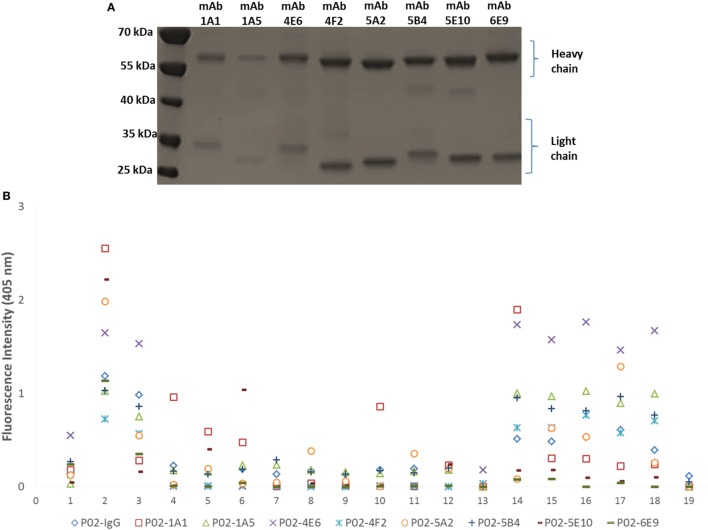
Characterization of patient-derived human monoclonal antibodies (hmAbs). **(A)** SDS-PAGE analysis of ~2 μg of purified hmAbs. **(B)** Reactivity of hmAbs was performed using formalin-fixed bacterial cells normalized to an OD600 of ~0.5. A 1:1,000 dilution of each hmAb was employed. A representative dataset from three biological replicates is presented. The coating antigens (protein antigens or cells) are represented by numbers on the *x*-axis as follows: 1. 4CMenB; 2. M14-240312; 3. MC58; 4. M07-240646; 5. M07-240657; 6. M07-240669; 7. M07-240679; 8. M07-240680; 9. M07-240909; 10. M08-240014; 11. M08-240107; 12. M08-240164; 13. M08-240276; 14. M10-240474; 15. M10-240476; 16. M10-240480; 17. M11-240016; 18. M11-240123; 19. *A. pleuropneumoniae*.

Amplification of the V_H_ and V_L_ (κ or λ) gene segments from 336 plasmablasts occurred at a PCR efficiency of 80%. Sequencing of these variable region gene segments showed biased usage of the IGHV3-7:IGλV2-8 gene pair, accounting for 16.7% of V gene usage. Of 139 recombinant hmAbs that were successfully expressed in HEK-293 cells, eight (Figure 3A) were reactive with the infecting MenB strain (M14-240312) and, in varying degrees, with the other members of the MenB strain panel (Figure 3B). None of these eight hmAbs were reactive with *Actinobacillus pleuropneumoniae* cells (a porcine respiratory pathogen). All antimeningococcal hmAbs possessed the IGHV3 class gene, albeit with different subclasses and light chain gene pairs; the exception being P02-4F2 (IGHV4-59:IGκV1/1D-39). Two of these hmAbs (P02-5E10 and P02-6E9), however, possessed identical V gene pairs (IGHV3-30:IGκV4-1).

**Figure 4:**
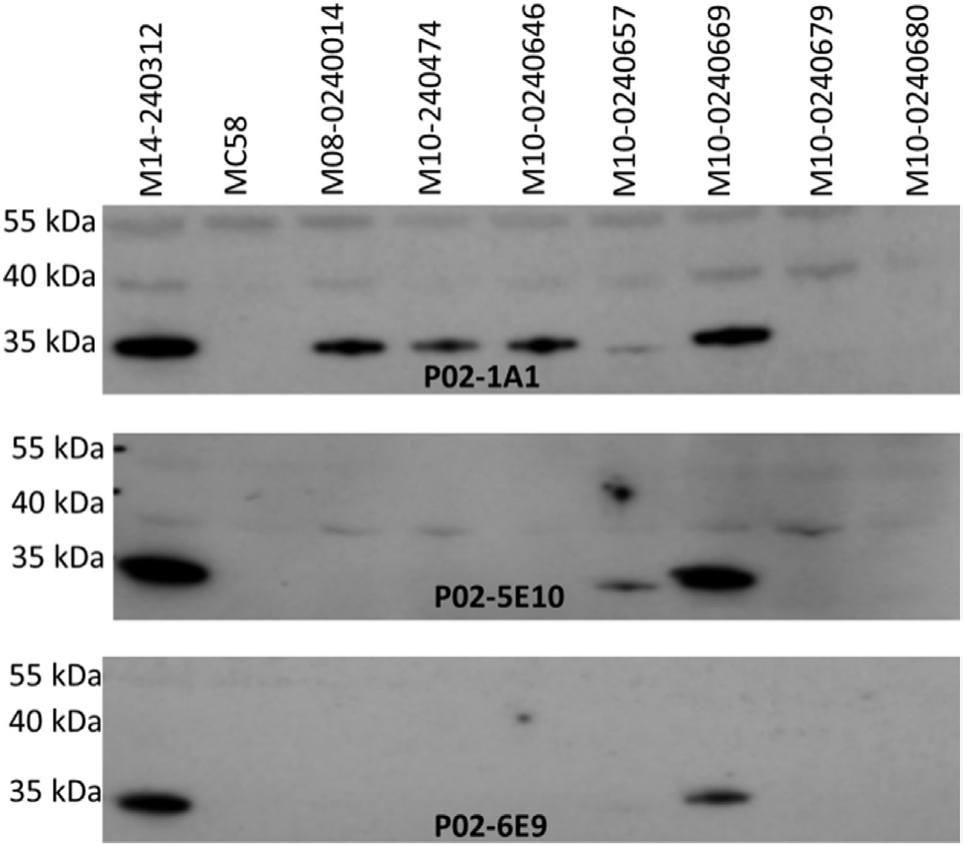
Specificity of patient-derived antimeningococcal (hmAbs) for linear epitopes in denaturing western blots. Lysates from normalized suspensions of selected MenB strains were used as template in western blot experiments. A 1:1,000 dilution of each hmAb was employed.

Specificity of the antimeningococcal hmAbs for the serogroup B capsule was ruled-out using the periodate assay, described in Ref. (35) (data not shown). Denaturing western blot data showed highly specific reactivity of three hmAbs (P02-1A1, P02-5E10, and P02-6E9) with a ~35 kDa meningococcal protein present in six members of the 17-strain panel (~35%). The target epitope of hmAb P02-1A1 was present in more strains (*n* = 6) than those of hmAbs P02-5E10 (*n* = 3) and P02-6E9 (*n* = 2) (Figure 4). The other five hmAbs, which were broadly cross-reactive with the strain panel (collectively recognizing 12 out of 17 strains), were non-reactive in denaturing western blots.

### Cloned Antimeningococcal hmAbs Possess SBA Activity Against Patient Isolate M14-240312

**Figure 5:**
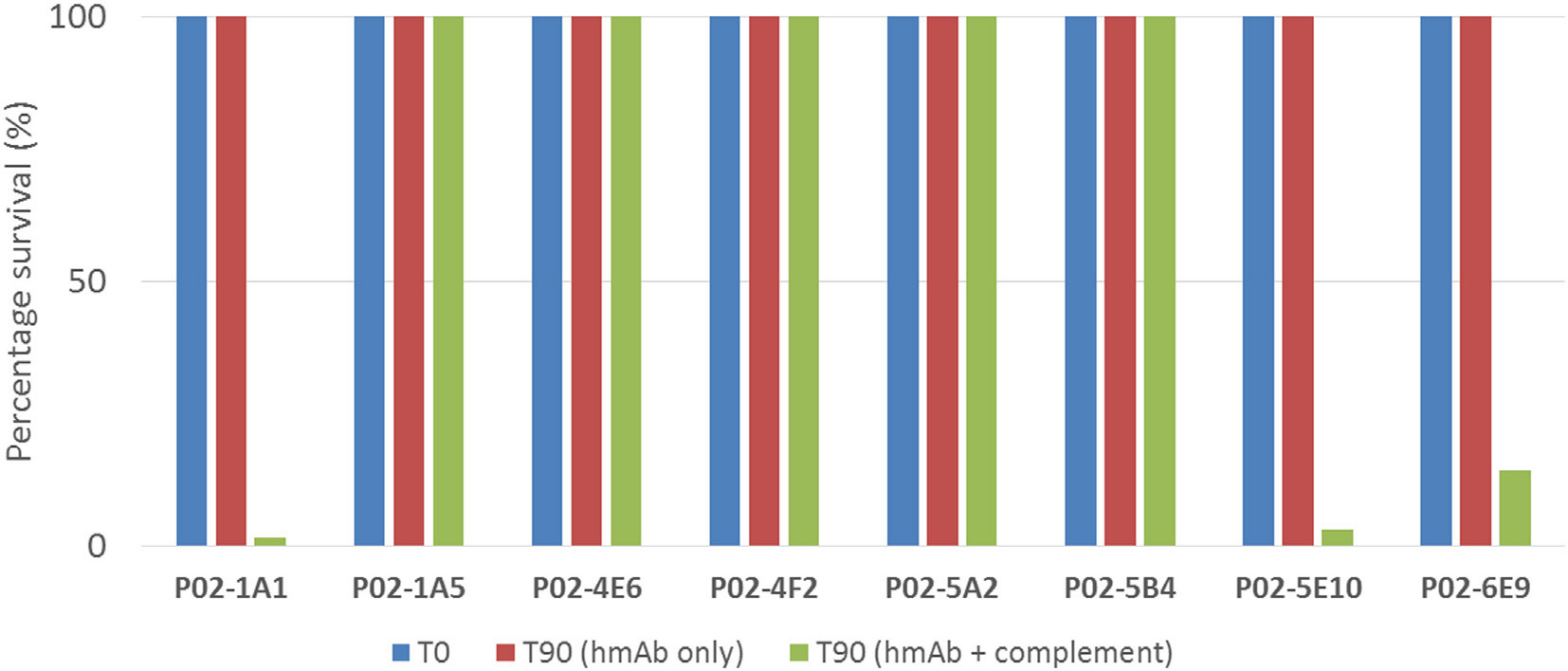
SBA of patient-derived antimeningococcal human monoclonal antibodies (hmAbs) versus patient isolate, M14-240312. Assays were performed with hmAbs normalized to final assay concentration of 80 µg/ml, in three biological replicates. Percentage survival after 60-min incubation (T60) in the presence of 25% human complement versus inoculum (T0) is shown.

To assess the functional activity of cloned recombinant hmAbs, SBAs were performed using purified hmAbs at a final assay concentration of 80 µg/ml. Three hmAbs (P02-1A1, P02-5E10, and P02-6E9), in synergy with exogenous human complement, possessed SBA activity against strain M14-240312. All other hmAbs were non-bactericidal (Figure 5).

### SBA Activity of hmAbs Is Strongly Linked to Surface Expression of Target Epitopes

To assess the breadth of SBA exhibited by hmAbs P02-1A1, P02-5E10, and P02-6E9, each hmAb was assayed for bactericidal activity against the 17-strain panel. HmAb P02-1A1-mediated killing of the patient isolate, M14-240312, and four other strains to which it bound in immunoassays (M07-240646, M07-240657, M08-240014, and M10-240474). Only one strain, M07-240669, which showed positive reactivity with hmAb P02-1A1 in immunoassays was resistant to SBA activity of P02-1A1.

**Figure 6:**
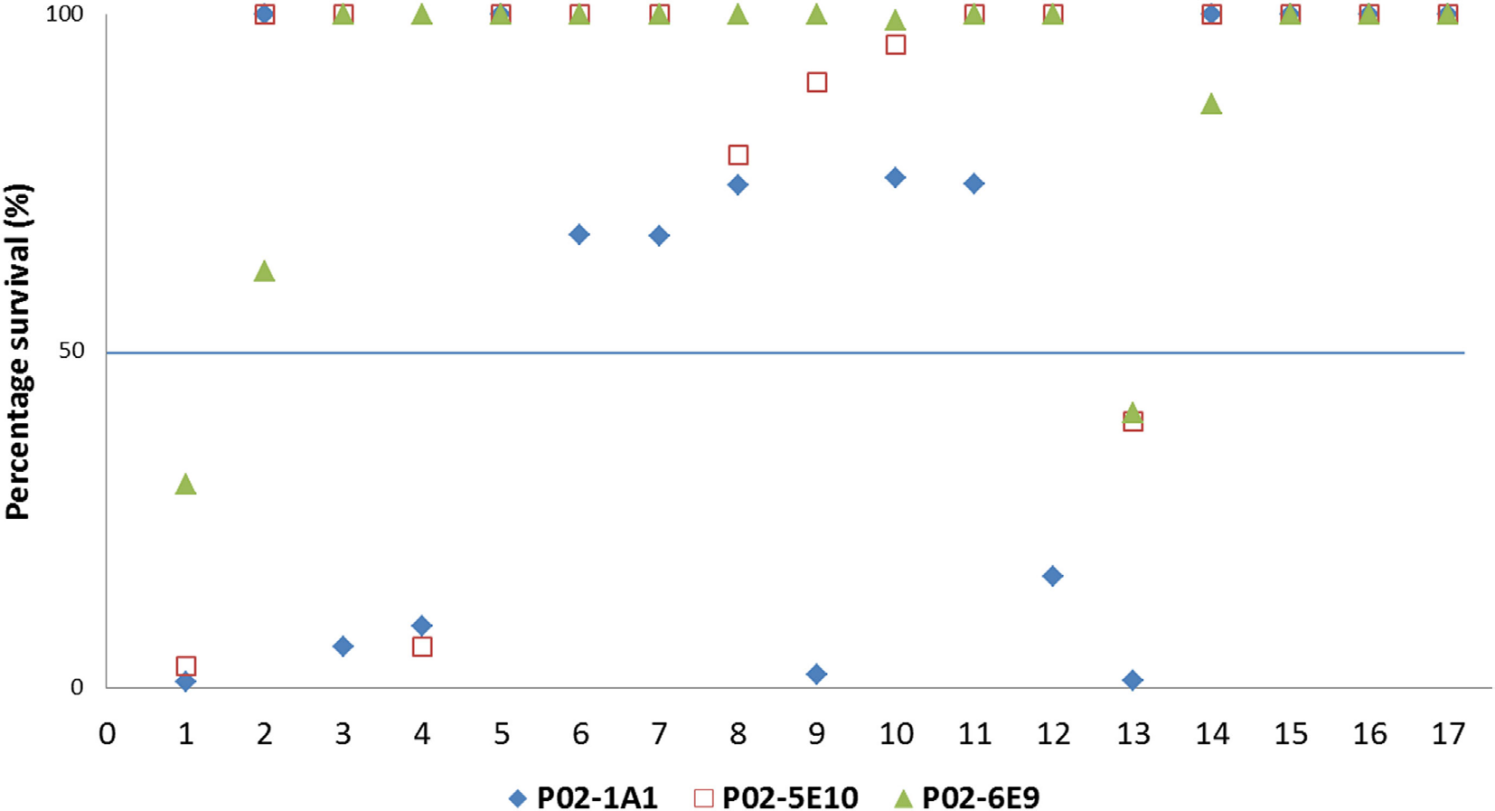
SBA of P02-1A1, P02-5E10, and P02-6E9 versus MenB strains. Numbers represent strains as follows: 1. M14-240312; 2. MC58; 3. M07-0240646; 4. M07-0240657; 5. M07-0240669; 6. M07-0240679; 7. M07-0240680; 8. M07-0240909; 9. M08-0240014; 10. M08-0240107; 11. M08-0240164; 12. M08-0240276; 13. M10-240474; 14. M10-240476; 15. M10-240480; 16. M11-240016; 17. M11-240123. Assays were performed with hmAbs normalized to a final assay concentration of 80 µg/ml, in three biological replicates.

Consistent with results obtained with P02-1A1, hmAb P02-5E10 possessed SBA activity against strain M10-240474 (to which it bound in immunoassays) but not strain M07-240669, suggesting resistance of M07-240669 to complement-dependent killing. Interestingly, all three bactericidal hmAbs could mediate killing of other strains to which no discernible reactivity was found in immunoassays; hmAb P02-1A1 reproducibly mediated killing of strain M08-240276 while both P02-5E10 and P02-6E9 mediated killing of strain M10-240474 (Figure 6; Table 2).

**Table 2:** Summary table showing reactivity of the bactericidal human monoclonal antibodies—P02-1A1, P02-5E10, and P02-6E9—in immunoassays (ELISA and western blot), and SBA activity versus the 17-strain MenB panel.

S/N	Isolate	P02-1A1		P02-5E10		P02-6E9	
		I	SBA	I	SBA	I	SBA
1	M14-240312	+	+	+	+	+	+
2	MC58	–	–	–	–	+	–
3	M07-240646	+	+	–	–	–	–
4	M07-240657	+	+	+	+	–	–
5	M07-240669	+	–	+	–	+	–
6	M07-240679	–	–	–	–	–	–
7	M07-240680	–	–	–	–	–	–
8	M07-240909	–	–	–	–	–	–
9	M08-240014	+	+	–	–	–	–
10	M08-240107	–	–	–	–	–	–
11	M08-240164	–	–	–	–	–	–
12	M08-240276	–	+	–	–	–	–
13	M10-240474	+	+	–	+	–	+
14	M10-240476	–	–	–	–	–	–
15	M10-240480	–	–	–	–	–	–
16	M11-240016	–	–	–	–	–	–
17	M11-240123	–	–	–	–	–	–

*I, immunoassay (ELISA and/or western blotting); SBA, serum bactericidal assay*.

*Plus (+) denotes positive reactivity in immunoassays or bactericidal activity in SBA*.

*Dash (–) denotes no discernible reactivity in immunoassays or bactericidal activity in SBA*.

## Discussion

Despite vaccination, the ongoing threat from IMD is unquestionable, especially in the African meningitis belt, justifying the search for novel approaches to vaccine candidate discovery. Reverse vaccinology 2.0 has been useful in understanding the human adaptive immune response to disseminated viral (36, 37) and bacterial infections (38, 39). Hence, it presents as a potentially powerful tool that may identify novel meningococcal vaccine candidate antigens or reinforce the candidacy of some known antigens.

In the present study, we succeeded in isolating functional hmAbs from a 7-month-old patient convalescing from IMD despite challenging practical issues. The most affected age group for IMD is 6- to 24-months, and patients presenting at the St. Mary’s PICU were mostly in this age group—children from whom the availability of sufficient quantities of blood sample for the isolation of PBMCs was very limited (4 ml). The isolation of specific antimeningococcal plasmablasts was further complicated by a paucity of knowledge on the magnitude and timing of peak plasmablast responses following primary meningococcal infection in infants. The plasmablast population measured in the single sample obtained at 7 days post-admission analyzed in this study was 0.5%. Plasmablast population following acute infection with dengue virus (37) occurred at 6–7 days postinfection. While the induction of peak plasmablast response following infection with nosocomially acquired bacteria such as *Acinetobacter baumanii* occurred in most patients at 8–16 days with 40–80% of the total B-cell population being plasmablasts, they composed >2% of B-cell population in patient samples obtained at 0–7 days postinfection (38). The low plasmablast induction seen in IMD patient SM-P02 may, therefore, be patient-specific and the timing of the peak level of cycling plasmablasts was likely missed (less than or more than 7 days post-admission). It is also plausible that low plasmablast induction is characteristic of a population at particular risk of IMD. Larger patient cohorts will be required to investigate this theory. Optimization of the IgG-cloning technique to increase the number of antimeningococcal plasmablasts using a recently published Ig capture-based assay (40) should improve productivity of the approach.

Reactivity of the antimeningococcal hmAbs isolated in this study with heterologous MenB strains possessing disparate PorA types strongly suggests that the antigen target of the hmAbs is not PorA, the immunodominant meningococcal antigen. It is likely that the three bactericidal hmAbs are reactive with a similar linear epitope contained in a yet-to-be identified ca. 35 kDa antigen. This 35 kDa antigen is surface-expressed in ~35% of the MenB strains, with heterologous 4CMenB antigen types, employed in this study. It is pertinent to note, however, that the ~35% presence of the linear epitope is specific to hmAbs generated in this study, and the antigen which it composes may possess more immunogenic surface-exposed epitopes and exhibit a wider presence among MenB and other meningococcal strains. One of these bactericidal hmAbs, P02-1A1, is reactive with a more diverse strain panel and possesses a higher bactericidal titer than hmAbs P02-5E10 and P02-6E9. This could be a result of somatic hypermutation of the P02-5E10 and P02-6E9 antibodies leading to the production of hmAb P02-1A1 with enhanced binding efficiency (affinity and/or avidity). Determination of the unequivocal identities of these hmAbs and their reactivities/SBA with other serogroups is currently ongoing. Taken together, data generated so far on these bactericidal hmAbs (P02-1A1, P02-5E10, and P02-6E9) strongly suggest that their target is not PorA, fHbp, or any of the 4CMenB recombinant antigens signifying its novelty and most importantly, candidacy for inclusion in future vaccine preparations. It is acknowledged, however, that while our current data suggest that the 35 kDa antigen is absent from the NZ OMV component of 4CMenB, further work is required to determine its unequivocal absence. Given the need for protein antigens that would compose improved or entirely novel cross-serogroup antimeningococcal vaccines, data from this study show that reverse vaccinology 2.0 can be employed as a useful tool in identifying functionally immunogenic antimeningococcal antigens.

## Ethics Statement

Studies with human blood samples were approved by the London—Fulham Research Ethics Committee (Ref.: 11/LO/1982). Informed written consent was obtained from patients or their representatives in accordance with the Declaration of Helsinki. Patients were recruited following admission to the Imperial Healthcare (St. Mary’s Hospital) Paediatric Intensive Care Unit (PICU), London, UK.

## Author Contributions

Conceptualization: PL, JK, SN, and GS; investigation: FB and PL; writing—original draft: FB; writing—reviewing and editing: FB, PL, SK, SN, and GS.

## Conflict of Interest Statement

The authors declare that the research was conducted in the absence of any commercial or financial relationships that could be construed as a potential conflict of interest. The reviewer MP and handling Editor declared their shared affiliation.

## References

[1] Caugant DA. Genetics and evolution of *Neisseria meningitidis*: importance for the epidemiology of meningococcal disease. Infect Genet Evol (2008) 8(5):558–65. 10.1016/j.meegid.2008.04.002 18479979

[2] Thompson MJ Ninis N Perera R Mayon-White R Phillips C Bailey L Clinical recognition of meningococcal disease in children and adolescents. Lancet (2006) 367(9508):397–403. 10.1016/S0140-6736(06)68609-1 16458763

[3] van de Beek D de Gans J Tunkel AR Wijdicks EF Community-acquired bacterial meningitis in adults. N Engl J Med (2006) 354(1):44–53. 10.1056/NEJMra052116 16394301

[4] Borrow R Alarcón P Carlos J Caugant DA Christensen H Debbag R The global meningococcal initiative: global epidemiology, the impact of vaccines on meningococcal disease and the importance of herd protection. Expert Rev Vaccines (2017) 16(4):313–28. 10.1080/14760584.2017.1258308 27820969

[5] Harrison LH Trotter CL Ramsay ME. Global epidemiology of meningococcal disease. Vaccine (2009) 27:B51–63. 10.1016/j.vaccine.2009.04.063 19477562

[6] Chandran A Herbert H Misurski D Santosham M. Long-term sequelae of childhood bacterial meningitis: an underappreciated problem. Pediatr Infect Dis J (2011) 30(1):3–6. 10.1097/INF.0b013e3181ef25f7 20683377

[7] Edwards MS Baker CJ. Complications and sequelae of meningococcal infections in children. J Pediatr (1981) 99(4):540–5. 10.1016/S0022-3476(81)80250-8 7277093

[8] Kirsch EA Barton RP Kitchen L Giroir BP Pathophysiology, treatment and outcome of meningococcemia: a review and recent experience. Pediatr Infect Dis J (1996) 15(11):967–79. 10.1097/00006454-199611000-00009 8933544

[9] Kristiansen PA Diomandé F Ba AK Sanou I Ouédraogo AS Ouédraogo R Impact of the serogroup A meningococcal conjugate vaccine, MenAfriVac, on carriage and herd immunity. Clin Infect Dis (2012) 56(3):354–63. 10.1093/cid/cis892 23087396

[10] Trotter CL Andrews NJ Kaczmarski EB Miller E Ramsay ME. Effectiveness of meningococcal serogroup C conjugate vaccine 4 years after introduction. Lancet (2004) 364(9431):365–7. 10.1016/S0140-6736(04)16725-1 15276396

[11] Villena R Safadi MAP Valenzuela MT Torres JP Finn A O’Ryan M. Global epidemiology of serogroup B meningococcal disease and opportunities for prevention with novel recombinant protein vaccines. Hum Vaccin Immunother (2018) 14(5):1042–57. 10.1080/21645515.2018.1458175 29667483 PMC5989912

[12] Edge C Waight P Ribeiro S Borrow R Ramsay M Ladhani S. Clinical diagnoses and outcomes of 4619 hospitalised cases of laboratory-confirmed invasive meningococcal disease in England: linkage analysis of multiple national databases. J Infect (2016) 73(5):427–36. 10.1016/j.jinf.2016.07.016 27475788

[13] Finne J Leinonen M Mäkelä PH. Antigenic similarities between brain components and bacteria causing meningitis: implications for vaccine development and pathogenesis. Lancet (1983) 322(8346):355–7. 10.1016/S0140-6736(83)90340-9 6135869

[14] Serruto D Bottomley MJ Ram S Giuliani MM Rappuoli R. The new multicomponent vaccine against meningococcal serogroup B, 4CMenB: immunological, functional and structural characterization of the antigens. Vaccine (2012) 30:B87–97. 10.1016/j.vaccine.2012.01.033 22607904 PMC3360877

[15] Parikh SR Andrews NJ Beebeejaun K Campbell H Ribeiro S Ward C Effectiveness and impact of a reduced infant schedule of 4CMenB vaccine against group B meningococcal disease in England: a national observational cohort study. Lancet (2016) 388(10061):2775–82. 10.1016/S0140-6736(16)31921-3 28100432

[16] Delrieu I Yaro S Tamekloe TA Njanpop-Lafourcade BM Tall H Jaillard P Emergence of epidemic *Neisseria meningitidis* serogroup X meningitis in Togo and Burkina Faso. PLoS One (2011) 6(5):e19513. 10.1371/journal.pone.0019513 21625480 PMC3098835

[17] Soeters HM Yameogo I Sawadogo G Aké F Lingani C Wang X Bacterial meningitis epidemiology and return of *Neisseria meningitidis* serogroup A cases in Burkina Faso in the five years following MenAfriVac mass vaccination campaign. PLoS One (2017) 12(11):e0187466. 10.1371/journal.pone.0187466 29095907 PMC5667755

[18] Sidikou F Zaneidou M Alkassoum I Schwartz S Issaka B Obama R Emergence of epidemic *Neisseria meningitidis* serogroup C in Niger, 2015: an analysis of national surveillance data. Lancet Infect Dis (2016) 16(11):1288–94. 10.1016/S1473-3099(16)30253-5 27567107 PMC5737706

[19] Trotter CL Lingani C Fernandez K Cooper LV Bita A Tevi-Benissan C Impact of MenAfriVac in nine countries of the African meningitis belt, 2010–15: an analysis of surveillance data. Lancet Infect Dis (2017) 17(8):867–72. 10.1016/S1473-3099(17)30301-8 28545721

[20] Gray SJ Trotter CL Ramsay ME Guiver M Fox AJ Borrow R Epidemiology of meningococcal disease in England and Wales 1993/94 to 2003/04: contribution and experiences of the Meningococcal Reference Unit. J Med Microbiol (2006) 55(7):887–96. 10.1099/jmm.0.46288-0 16772416

[21] Halperin SA Bettinger JA Greenwood B Harrison LH Jelfs J Ladhani SN The changing and dynamic epidemiology of meningococcal disease. Vaccine (2012) 30:B26–36. 10.1016/j.vaccine.2011.12.032 22178525

[22] Whittaker R Dias JG Ramliden M Ködmön C Economopoulou A Beer N The epidemiology of invasive meningococcal disease in EU/EEA countries, 2004–2014. Vaccine (2017) 35(16):2034–41. 10.1016/j.vaccine.2017.03.007 28314560

[23] Mowlaboccus S Perkins TT Smith H Sloots T Tozer S Prempeh LJ Temporal changes in BEXSERO^®^ antigen sequence type associated with genetic lineages of *Neisseria meningitidis* over a 15-year period in Western Australia. PLoS One (2016) 11(6):e0158315. 10.1371/journal.pone.0158315 27355628 PMC4927168

[24] DeKosky BJ Ippolito GC Deschner RP Lavinder JJ Wine Y Rawlings BM High-throughput sequencing of the paired human immunoglobulin heavy and light chain repertoire. Nat Biotechnol (2013) 31(2):166. 10.1038/nbt.2492 23334449 PMC3910347

[25] Georgiou G Ippolito GC Beausang J Busse CE Wardemann H Quake SR. The promise and challenge of high-throughput sequencing of the antibody repertoire. Nat Biotechnol (2014) 32(2):158. 10.1038/nbt.2782 24441474 PMC4113560

[26] Rappuoli R Bottomley MJ D’Oro U Finco O De Gregorio E. Reverse vaccinology 2.0: human immunology instructs vaccine antigen design. J Exp Med (2016) 213(4):469–81. 10.1084/jem.20151960 27022144 PMC4821650

[27] Schieffelin JS Costin JM Nicholson CO Orgeron NM Fontaine KA Isern S Neutralizing and non-neutralizing monoclonal antibodies against dengue virus E protein derived from a naturally infected patient. Virol J (2010) 7(1):28. 10.1186/1743-422X-7-28 20132551 PMC2829534

[28] Wrammert J Smith K Miller J Langley WA Kokko K Larsen C Rapid cloning of high-affinity human monoclonal antibodies against influenza virus. Nature (2008) 453(7195):667. 10.1038/nature06890 18449194 PMC2515609

[29] Wu X Yang ZY Li Y Hogerkorp CM Schief WR Seaman MS Rational design of envelope identifies broadly neutralizing human monoclonal antibodies to HIV-1. Science (2010) 329(5993):856–61. 10.1126/science.1187659 20616233 PMC2965066

[30] Fink K. Origin and function of circulating plasmablasts during acute viral infections. Front Immunol (2012) 3:78. 10.3389/fimmu.2012.00078 22566959 PMC3341968

[31] Smith K Garman L Wrammert J Zheng NY Capra JD Ahmed R Rapid generation of fully human monoclonal antibodies specific to a vaccinating antigen. Nat Protoc (2009) 4(3):372. 10.1038/nprot.2009.3 19247287 PMC2750034

[32] Kohl TO Ascoli CA Direct and indirect cell-based enzyme-linked immunosorbent assay. Cold Spring Harb Protoc (2017) 2017(5):402–7. 10.1101/pdb.prot093757 28461659

[33] Bidmos FA Chan H Praekelt U Tauseef I Ali YM Kaczmarski EB Investigation into the antigenic properties and contributions to growth in blood of the meningococcal haemoglobin receptors, HpuAB and HmbR. PLoS One (2015) 10(7):e0133855. 10.1371/journal.pone.0133855 26208277 PMC4514712

[34] Borrow R Aaberge IS Santos GF Eudey TL Oster P Glennie A Interlaboratory standardization of the measurement of serum bactericidal activity by using human complement against meningococcal serogroup B, strain 44/76-SL, before and after vaccination with the Norwegian MenBvac outer membrane vesicle vaccine. Clin Diagn Lab Immunol (2005) 12(8):970–6. 10.1128/CDLI.12.8.970-976.2005 16085915 PMC1182195

[35] Woodward MP Young WW Jr Bloodgood RA. Detection of monoclonal antibodies specific for carbohydrate epitopes using periodate oxidation. J Immunol Methods (1985) 78(1):143–53. 10.1016/0022-1759(85)90337-0 2580026

[36] Goodwin E Gilman MS Wrapp D Chen M Ngwuta JO Moin SM Infants infected with respiratory syncytial virus generate potent neutralizing antibodies that lack somatic hypermutation. Immunity (2018) 48(2):339–49. 10.1016/j.immuni.2018.01.005 29396163 PMC6005179

[37] Wrammert J Onlamoon N Akondy RS Perng GC Polsrila K Chandele A Rapid and massive virus-specific plasmablast responses during acute dengue virus infection in humans. J Virol (2012) 86(6):2911–8. 10.1128/JVI.06075-11 22238318 PMC3302324

[38] Band VI Ibegbu C Kaur SP Cagle SM Trible R Jones CL Induction of human plasmablasts during infection with antibiotic-resistant nosocomial bacteria. J Antimicrob Chemother (2014) 69(7):1830–3. 10.1093/jac/dku047 24583361 PMC4054983

[39] Zimmermann N Thormann V Hu B Köhler AB Imai-Matsushima A Locht C Human isotype-dependent inhibitory antibody responses against *Mycobacterium tuberculosis*. EMBO Mol Med (2016) 8(11):1325–39. 10.15252/emmm.201606330 27729388 PMC5090662

[40] Pinder CL Kratochvil S Cizmeci D Muir L Guo Y Shattock RJ Isolation and characterization of antigen-specific plasmablasts using a novel flow cytometry-based Ig capture assay. J Immunol (2017) 199(12):4180–8. 10.4049/jimmunol.1701253 29118244 PMC5720343

